# Meaning-Making Through Creativity During COVID-19

**DOI:** 10.3389/fpsyg.2020.595990

**Published:** 2020-12-18

**Authors:** Hansika Kapoor, James C. Kaufman

**Affiliations:** ^1^Monk Prayogshala, Mumbai, India; ^2^Neag School of Education, University of Connecticut, Storrs, CT, United States

**Keywords:** adaptive response, COVID-19, creativity, meaning-making, pandemic

## Abstract

The COVID-19 pandemic resulted in an abrupt change in routines and livelihoods all around the world. This public health crisis amplified a number of systemic inequalities that led to populations needing to grapple with universally difficult truths. Yet some individuals, firms, and countries displayed resilient and creative responses in coping with pressing demands on healthcare and basic sanity. Past work has suggested that engaging in creative acts can be an adaptive response to a changing environment. Therefore, the purpose of this paper is to describe how entities at the personal, community, and national levels cultivated and expressed creativity in an effort to make meaning during COVID-19. By overlaying the Four C model of creativity on such responses, we aim to (a) to connect mini, little, Pro, and Big creative behaviors with our attempts to make meaning of the ongoing COVID-19 pandemic, and (b) to suggest how engaging in creative expression can be used to guard against the adverse consequences of this outbreak. Acknowledging that this time has been and continues to be distressing and filled with uncertainty, we propose some ways of making sense of current events by applying original thinking across domains. Further, we propose how engaging in creativity can serve to buffer against the negative effects of living through the pandemic.

“It is not the strongest of the species that survives, nor the most intelligent. It is the one that is the most adaptable to change.”

– *Charles Darwin*

The ongoing COVID-19 pandemic has resulted in 1.4 million deaths and over 58 million infections worldwide at the time of writing (Dong et al., 2020). These numbers are on the rise, as is the resultant socioeconomic discord across the globe. This includes criticism of local and national government policies in response to the pandemic and adverse economic consequences of halting trade and transport between nations. The year 2020 has been upsetting not only at the global level, but also closer to home—with individuals, families, and communities bearing the brunt of changes in daily routines and habits. These disruptions are because some of the primary ways to contain the spread of the virus are by maintaining physical distance from others, regular hand-washing, and wearing masks in public. The pandemic has thus had a great deal of collateral damage, such as stress and fear, even for people who have not become ill (Pfefferbaum and North, 2020). It is unlikely that we will see major positive changes any time soon.

What does this worldwide event mean in the grand scheme of our lives? Could it plausibly be a paradigm shift in how humanity views itself and the world or are we waiting for a vaccine to be developed so that things can return to normal? What does it mean to lose a loved one over the phone, without the ability to be by their bedside because of the risk of contracting the virus in that short span? During such times, it is natural to seek out meaning in life. There are many pathways and sources of finding such meaning; one such example is through the act of creation and engagement in creative pursuits (see also Sligte et al., 2013). Therefore, the two chief aims of the present work are (a) to connect different levels of creative behaviors with our attempts to make meaning of the ongoing COVID-19 pandemic, and (b) to suggest how engaging in creativity can be used to shield against the negative consequences of this outbreak. An ancillary objective is to document the scope and kinds of originality displayed by laypersons and experts alike during this historical time.

## Meaning-Making and Creativity

Many modern theories of meaning-making have their roots in classic humanist scholars from many decades ago. Maslow’s (1943) hierarchy of needs culminates in self-actualization, in which a person is able to fulfill their potential. There are many ways one can achieve this peak; one path is by utilizing personal abilities, which can easily include maximizing creativity. Frankl’s (1946) approach to how people find meaning in their lives derived from his experiences in concentration camps. He proposed three ways that people can achieve meaning: (a) creating or completing a particular task or work; (b) through an experience or interpersonal connection; and (c) how people face unavoidable pain and suffering. Lifton (1979, 2011) studied both survivors and perpetrators of wartime evil and focused on the idea of symbolic immortality. We are all mortal and must face the prospect of an inevitable death. As a result, one way we cope is by seeking out symbolic immortality, or a way of living on even after we die. Lifton proposed five ways that this path can be reached: (a) having children; (b) focusing on links to the past and future through our physical matter; (c) turning to spirituality or religion; (d) deciding to live each moment to its fullest possible experience; and (e) emphasizing the impact of one’s work, mentorship of others, and creative output. The role of creativity in finding meaning is both explicitly stated and implicitly found in many other components of both Frankl’s and Lifton’s conceptions.

More recently, Martela and Steger (2016) propose that there are three key aspects of meaning: Coherence, significance, and purpose. Coherence is being able to make sense of one’s life, as opposed to seeing the past as being a series of random and chaotic events. Significance is seeing value, joy, and connection in one’s everyday life. Purpose is having plans and goals for the future. Kaufman (2018) highlighted many ways in which creativity can serve all three dimensions of this conception of meaning. It is important to note that across all of these (and other) models, creativity is but one way to achieve meaning. Spirituality, empowerment, and benevolence toward others are equally viable pathways depending on the individual (e.g., Bailey et al., 2019).

One way to enhance coherence is to engage in what is often called the “writing cure” (Pennebaker and Beall, 1986; Pennebaker, 1997). This process entails writing expressively several times a week about personal, emotional topics. This writing does not necessarily need to be creative in nature (it could be keeping a journal, for example), but it needs to have some element of a narrative (Pennebaker and Seagal, 1999). People who write under these broad guidelines have been shown to have notable benefits in both physical and mental health (Travagin et al., 2015). In general, writing a memoir about one’s past has often been used as a tool in therapy (Riessman, 2003).

Creativity can help people reach significance in many ways. Most strikingly, the act of being creative can be enjoyable and enthralling all on its own, regardless of any specific outcome. Consider Csikszentmihalyi’s (1996) concept of Flow, in which one is actively engaged in a pleasurable pursuit. These can also include athletics or other favorite hobbies, but creative undertakings are a common way to enter Flow. This sensation of being immersed in something creative, often losing track of time and one’s surroundings, can be intensely joyful. Living a life filled with such pleasures is one way to enhance significance. Similarly, art-making has been shown to improve one’s mood; as opposed to the mechanisms of the “writing cure,” art-making helps because it is fun and distracting (Drake and Winner, 2012, 2013). Finally, experiencing creative works such as art in the shared presence of other people (i.e., in a museum) increases feelings of connection (Smith, 2014).

Creativity can help people feel increased purpose in a variety of ways. Creative writing can help people articulate better career narratives that can help them strive for more meaning in their work (Lengelle et al., 2016). Continued generativity across the lifespan can help prevent people from feeling stagnant (McAdams et al., 1993). Further, the need to leave a legacy, similar to Lifton’s (1979) symbolic immortality, can lead people to pass along their creativity in any form, whether to family members and friends or to the world at large.

In these times of uncertainty and change, the need to find meaning is even more salient. During this pandemic, several aspects seem uncertain and undetermined: from who catches the virus and the extent of its physical impact to each country or region’s changing response. Consequently, the need for coherence in one’s life becomes essential. As lockdown has continued to be in effect around many parts of the world, our interpersonal connections have often been weakened and daily moments of joy or value may feel rare. We need to find significance more than ever. Finally, with an unclear future and both short- and long-term uncertainty, finding purpose is crucial to moving forward.

## The Four Cs During COVID-19

In this context and beyond, a useful taxonomy to analyze creativity is the Four C model (Beghetto and Kaufman, 2007; Kaufman and Beghetto, 2009). This framework proposes a developmental trajectory that can play out over one’s lifespan or over one’s activity in a particular domain. It begins with *mini-c*, which categorizes explorative behavior that may not necessarily be novel in a larger, historical sense, but is personally meaningful to the individual creator. For instance, when a child creates a secret handshake to be used only with certain others, they display mini-c creativity. *Little-c*, or everyday creativity, comprises those actions that most of us engage in on a regular basis, such as finding new ways to redecorate one’s home or coordinating mix-and-match outfits. Both mini- and little-c creativity are manifested by non-specialists. The next category of *Pro-c* is reserved for those who are approaching or have reached expert-level creativity, but may not have achieved eminence yet. A professional chef at a local restaurant would classify as Pro-c. If this individual makes an innovative cooking contribution (such as creating a classic dish) that people continue to enjoy for years after their death, then they can be considered to have progressed to the last category of *Big-C* creativity. Often regarded as geniuses in their domain, Big-C creators can represent the peak of what may be achieved.

In the context of COVID-19, the Four C model provides a systematic structure to analyze the surge of original behavior, both online and offline. Mini-c and little-c behaviors are displayed at the personal level and are intended to assist with coping, distraction, or simply finding amusement at a time like this. Such endeavors can be common; one experience-sampling study indicated that people were in the process of doing something creative nearly 22% of the time (Silvia et al., 2014). Past research has suggested that partaking in everyday creative activities is associated with positive affect and well-being (Forgeard et al., 2014; Richards, 2007). In light of the pandemic, it is possible that this frequency has increased as a means to cope with current uncertainties and insecurities, enabling one to seek and find significance in the mundane (Martela and Steger, 2016). Moreover, a principal motive for everyday creativity was enjoyment (Benedek et al., 2019), something that became a scarce experience when lockdowns were enforced.

In the initial months of the quarantine period (ranging from January to April 2020 across different countries), social media became an especially popular platform for expressing such creativity. For instance, a 9-second video of a sock puppet eating up cars in moving traffic (@gnuman1979) was posted on March 16, 2020 and went on to accumulate more than 3 million likes^1^; the caption was plainly, “Quarantine Day 6.” The post was one of the first of a number of other original videos of people trying to maintain their sanity by performing regular or whimsical activities, despite the lockdown. For instance, another post (@NigheanMo) showed a man using a combination of dish soap and water to create a makeshift treadmill against the kitchen counter^2^. Other posts, such as those of parents trying new ways to keep their children busy while they worked from home or people trying to play tennis across windows or rooftops, also represented everyday creativity.

Another trend that rapidly went viral was individuals dressing up in Halloween and other costumes to take out the trash. “Bin Isolation Outing” became an accepted way to have some fun when disposing of garbage and simultaneously meeting the requirement of wearing a mask or face covering when outdoors. Using toothpicks to press elevator buttons or covering light switches with saran wrap were other creative ways to restrict touching surfaces in public spaces. People with basic sewing skills started making homemade masks so that the supply of essential PPE (Personal Protective Equipment) could be reserved for healthcare workers. Residents in Italy, one of the earliest and hardest hit countries in the pandemic, took to singing from their balconies to maintain solidarity in difficult times. A few UPS employees took to dressing up as superheroes to bring some cheer into their customers’ (and presumably their own) lives. Across the world, appreciation for those on the frontlines of the fight against COVID-19 was displayed through applause, sirens, honking, and generally making a din. Such demonstrations allowed people to express their gratitude while still staying sheltered, thus representing an original use of space and sound. Moreover, these creative behaviors are recorded and disseminated via social media, accessible to most across the world; potentially, with enough exposure and appreciation, some of these little-c activities may become Pro-c. Even if not, these original nuggets along with this paper could be viewed as a way to make coherent sense of the chaotic twists our lives have taken in the past months.

Those employed in creative professions also took to innovatively using their skills during the pandemic expressing Pro-c abilities. Several professional musicians provided free online home concerts in an effort to raise money for relief work through donations. All types of artists took to offering online classes to help at-home parents entertain their children. Healthcare workers also joined in by (unfortunately) having to innovate their PPE in the absence of adequate supplies. The video of an Olympic swimmer (@SvRouwendaal) innovatively using a bungee cord to develop resistance as she trained in a kiddie pool made the rounds on social media as well^3^; it exemplified the back-and-forth between the Cs, wherein a professional sportsperson engaged in everyday creativity to solve the problem of staying active during the lockdown. Any CEO of a business, large or small, was compelled to adapt to changing circumstances to keep from going under. Some professional chefs volunteered to cook and distribute meals to those in need. In terms of scientific creativity, researchers began working toward developing a vaccine against this novel coronavirus as soon as its genetic sequence became available in February 2020 (Ren et al., 2020). The pandemic also brought together the global scientific community, paving novel ways for collaborations and partnerships to defeat a common enemy. Moreover, behavioral researchers took to conducting surveys and using social science to help enforce social distancing norms across the world (e.g., Tagat and Kapoor, 2020). The need to make meaning and find purpose in an uncertain and confusing situation using one’s professional skills was observed in some manner across the board.

The significance of the creative economy and of cultural industries came to the fore early on during the pandemic. Laypeople turned to the creative arts to seek solace and make sense of the ongoing crisis. The concerns of those in creative occupations, although longstanding, became much more visible owing to the COVID-19 outbreak (Comunian and England, 2020). Yet, a wide variety of experts across the globe persisted in resiliently displaying their Pro-c across diverse occupations, from teachers to late-night comedians. The former had to creatively adapt to using technology in the virtual classroom, whereas the latter had to build workarounds for taping shows from their homes and learn how to deliver material in the absence of feedback from a live studio audience.

To reiterate, those who achieve eminent status as a result of their professional creative endeavors may possibly go on to reach Big-C. Usually, this transition is determined by the passage of time and by the reputation earned by the individual over the course of their lifetime (and beyond). However, at a time like the current pandemic, there are some individuals who have displayed what could end up being considered Big-C creativity across the mere span of months. Similarly, there are others who may be at the cusp of achieving Big-C eminence based on their response to the pandemic. The obvious frontrunners in the latter category are the teams of scientists (all currently Pro-c) who are working on developing a vaccine against the novel coronavirus. For instance, prior to the current outbreak, researchers at the Oxford Vaccine Group were developing a vaccine for MERS (Middle Eastern Respiratory Syndrome). Clinical trials for this vaccine were underway when COVID-19 began spreading across the world; the researchers pivoted their agenda from developing a MERS vaccine to a COVID-19 vaccine, thus enabling them to gain a head start in the race for this novel virus (Masetti et al., 2020).

Among politicians and government officials, the efforts of Jacinda Ardern (Prime Minister of New Zealand) and Gavin Newsom (Governor of California) bear mention. Ardern was one of the first world leaders to enforce strict travel restrictions as well as state-mandated quarantine for incoming passengers, as early as February 2020. This move was implemented unapologetically to secure the safety of the country’s citizens. It was an early and effective step, with New Zealand’s COVID cases sharply declining since mid-April 2020. Ardern’s combination of strategies represent a highly appropriate and original response to the rapidly evolving situation. Similarly, Newsom responded to the crisis by facilitating an online marketplace where citizens requiring PPE could purchase it directly from California-based businesses who had pivoted their operations^4^. This move side-stepped arduous wait times for crucial and life-saving equipment needed by citizens and businesses alike. Such innovative and creative problem-solving is needed to deal with this public health crisis effectively (see also Cohen and Cromwell, 2020). Depending on the continued impact and historical view of their actions, they could end up as Big-C politicians.

Another potential contender for future Big-C status is Indian movie actor Sonu Sood. In March 2020, India responded to the ongoing spread of COVID-19 with a nationwide lockdown, halting national and international transportation nearly overnight. This action meant that millions of migrant laborers in metropolitan cities like Mumbai were stranded, with no source of income and no way to return home. Sood coordinated with state governments and transportation agencies to arrange special buses, trains, and flights to ensure that migrant workers and their families made their way to their respective home states. Sood initially sought such requests on Twitter and coordinated with passengers using the social media platform. He also launched a website called *Pravasi Rojgar* (Migrant Employment) that features job opportunities across India for the displaced workers. Sood’s coordinated and sustained effort to not only help migrants travel home, but also reintegrate them into the labor force was lauded across India. This humanitarian work may contribute as much to his legacy as the movies with which he has been associated.

Overall, we observed that as creative actions progressed across the Four Cs, individual-level behaviors expanded to community and even national level involvement. Although it is difficult to neatly delineate group creative processes from individual ones, it is likely that the former thrived when leaders encouraged innovative solutions (Paulus et al., 2012), be it in companies or governments. By design, collaborative creativity reduced during the pandemic when lockdown was enforced; for instance, musicians, actors, or other creative arts professionals were confined to their homes with little scope to jointly create, at least initially. As with everything else, such collaborations moved online, giving further credence to the role that creativity plays in achieving coherence, significance, and purpose (see also Kaufman, 2018).

## Creativity and Creation as a Buffer

By definition, creativity encompasses original and appropriate behaviors (Plucker et al., 2004; Runco and Jaeger, 2012). Yet, individuals amidst the pandemic were drawn not only to behaving creatively but engaging in the act of creation itself. For instance, the proliferation of people wanting to bake sourdough or make Dalgona coffee in April 2020 and onward was a prominent trend in middle to upper income households. However, this act is less likely to qualify as creative over a period of time because a central tenet is that creativity, namely originality, decays with the passage of time. Nonetheless, individuals continued to have the need to express or distract themselves through creating *something.* The very act of making something seems to have value by itself, possibly as a method to cope with uncertainty and tolerate ambiguity (see also Zenasni et al., 2008; Kornilova and Kornilov, 2010; Merrotsy, 2013). It is important to note that these needs can be met through production or activities that might not be seen as creative by other people. The concept of mini-c highlights how personal inspiration can be quite meaningful (Beghetto and Kaufman, 2007), and it is possible for people to be creative in ways that they themselves may not recognize (Kaufman and Glãveanu, 2020) or even be consciously aware (Cropley and Cropley, 2009).

Simultaneously, there has been a noticeable trend of people around the world consuming similar creative content. Those individuals with access to resources binge-watched similar programming (such as the Netflix documentary *Tiger King* or the Amazon series *Paatal Lok*) around the same time. Of course, some of the reason was that people were largely restricted to staying in, so any home-based activity was more likely to be pursued. Yet it is consistent with past studies of aesthetic appreciation (Smith, 2014) that we can feel connected to others by consuming the same creative content (and, often, discussing such works with each other online; Biasutti, 2015). For instance, viewing and listening parties have become a popular way to experience this connection virtually during the quarantine.

In a similar vein, research has identified associations between creativity and building resilience, particularly as a response to adversity (Metzl and Morrell, 2008; Metzl, 2009); as an important component of responding to disasters (Kendra and Wachtendorf, 2003); and as a facilitative process in achieving post-traumatic growth (Forgeard, 2013). Psychological resilience is mental armor against crises, resulting in successful adaptation to the current circumstance (Fletcher and Sarkar, 2013). Similarly, creativity is characterized by generating adaptive responses to respond to new conditions and environments (e.g., Cohen, 2012). Therefore, we argue that making meaning through any kind of creative expression is an adaptive and resilient response to the ongoing pandemic. This connection is particularly strong because the COVID-19 outbreak has made human morality incredibly salient. Past research has found that in the face of such existential crises, creativity can not only increase, but tends to be directed toward establishing a legacy (Routledge et al., 2008; Sligte et al., 2013; Kaufman, 2018).

The psychological consequences of the current pandemic are catastrophic, including generalized anxiety, depressed affect, insomnia, and fear across the world (e.g., Huang and Zhao, 2020; Shevlin et al., 2020; Tang et al., 2020; Torales et al., 2020). Although expressing oneself through creative actions is no silver bullet, such expression can build resilience and lessen the impact of current stressors. Research has identified how participatory arts projects can foster well-being in the community (Cameron et al., 2013), particularly in older populations (Liddle et al., 2013). Given current stressors—and their potential profound impact among the elderly, who are most at risk—it may be time to seek and implement similar innovative solutions to combat downturns in mental health and productivity. For instance, artist teams can set up workshops within online communities to facilitate artistic expression as well as promote healthy behaviors. Given that scholars on vulnerable older populations have pushed for exploring interventions beyond the arts (e.g., Bellass et al., 2019), this time may be particularly apt for such new directions.

However, the dark side of creativity at a time like this cannot be discounted (e.g., Cropley et al., 2010). Ranging from students downvoting homework applications to remove them from app stores (Cuthbertson, 2020) to fake coronavirus testing kits (Tahir, 2020) to rampant misinformation and conspiracy theories related to COVID-19 (e.g., Uscinski et al., 2020), the use of originality for self-gain has persisted. Without additional research, it would be premature to make assumptions about the extent to which such creativity fuels meaning-making for its actors; however, these examples may be coherent, significant, and purposeful in their own right, arising from an amoral creative process (Runco, 2010).

## Conclusion

COVID-19 is the largest natural experiment that humanity has been subjected to in recent times. The pandemic has amplified pre-existing inequalities the world over, including fragile healthcare systems and mismanaged funds. In the wake of this “experiment,” creativity under constraints has emerged (e.g., Haught-Tromp, 2017; Medeiros et al., 2018)—and how. Overall, people across all levels of creative accomplishment and ability have attempted and succeeded at responding to emerging challenges with a wide array of innovation and originality. In this paper, we argue that such creativity is an avenue to make meaning of current happenings. After all, it is second nature for us as a species to enjoy the presence of meaning as well as to seek out meaning in our lives (see also Li et al., 2020).

Future research can aim to identify relationships between engaging in creative actions and meaning-making empirically. A mixed-methods approach may be well-suited to answer the broad question of whether and how a creativity intervention can influence individuals’ sense of well-being and purpose in the midst of a pandemic. Moreover, as COVID-19 has had varying impacts across age groups, targeted studies can infer if creative thinking is a safeguard worth investigating. A vast amount of scholarship suggests using creative expression to cope better and achieve post-traumatic growth through artistic, literary, and similar endeavors (e.g., Pennebaker, 1997; Drake and Winner, 2012; Forgeard et al., 2014). Therefore, we propose that regularly engaging in some creative activity can be associated with improved well-being and coping during the pandemic. An example could be a creative expression intervention, documented using videos, storytelling, and photography; the task could involve uploading a picture or video of an object/scene at home and writing a story about the same, once a week over the course of 4 weeks.

At a broader level, Cameron et al. (2013) developed guidelines to organize participatory arts projects in local communities to promote well-being. Subsequent research can investigate whether using participatory creativity interventions can help design future activities and lead to reimagining of spaces, repurposing existing resources, bridging gaps in socioeconomic disparities, and the cultural landscape (e.g., Lewis, 2013). Such research can contribute to the existing literature in the areas of resilience, meaning-making, social identity, and post-traumatic growth.

We assume that a vaccine will eventually be discovered and at that time, we will have to decide how to move forward with establishing a new normal of fundamental human interaction, systems, operations, and processes. All our collective creative energy that has been released during the quarantine/lockdown may help shape this new normal, or else we may revert to pre-outbreak times with little to show for our lived experience (and shared trauma). The months between the declaration of COVID-19 as a pandemic and the mass rollout of a vaccine are ripe for creative solutions across domains; divergent thinking has dominated our problem-solving at this time. After the development of a vaccine, we would need to employ convergent thinking to then consolidate and evaluate the several solutions we have devised during this period. For instance, as social distancing is important to mitigate the spread of the infection, several activities have predominantly or entirely moved online. Virtual academic conferences, e-learning student activities, and educational webinars have mushroomed in the past few months, providing a sense of interactivity and knowledge sharing. The bright side of such virtual events is their increased accessibility to individuals across the globe, regardless of status, funding, or even time zone. There are, of course, many downsides as well, such as reduced personal interaction, decreased revenue for conference organizers (registration fees and overheads of online events are usually nominal in comparison with in-person standard fees), and the limits of passive learning.

What will happen once the pandemic is under control and it is safe to organize such events offline and in person again? Will virtual events continue to be offered in a hybrid model (with offline and online presentations) or will we revert to the way things were, with more restricted access to information and people? Will the regular online arts exchanges become part of our routines, or are they purely due to circumstance? It is a paradox that at such a dark time for the performing arts, when an astoundingly high percentage of workers in these professions are unemployed due the pandemic (Lang and Maddaus, 2020), it is also a moment when such performances have become accessible to more people than ever before. A watershed event such as Disney Plus screening the filmed *Hamilton* may have turned countless people onto the glory of theatre at a time when actual live theatre is largely impossible. The performing arts also can serve as an example for how to potentially transition lockdown learnings into a post-pandemic world. One reason why *Hamilton* was such a rarity is that in the past, shows have infrequently been publicly streamed because of complex nuances of determining royalties and rights. In the initial months of lockdown, copyright issues were largely overlooked. As streaming productions became more common and most theatres began to utilize this technique as a way of bringing in some revenue, the two unions for theatre and film actors came to an agreement. The theatre actors’ union can represent streaming work until December 31, 2021, as opposed to having actors in streaming productions also be required to join the film actors’ union (Meyer, 2020). If a similar type of arrangement can be put in place on a more permanent basis, online streaming theatre may continue to be commonplace after the virus is but a memory.

The idea to not let this creativity go to waste seems noble, yet this decision is to be taken collectively and collaboratively. Currently, online interactions are the best solution we have because it is simply not safe enough to travel or interact with others on a regular basis. Relatively few people, given an equal opportunity to pursue either option, would consider such online meetings to be superior to face-to-face interactions. However, we are now presented with an opportunity to modify such preferences, which can have meaningful consequences for the future. For instance, restricted travel has effectively reduced emissions, thereby impacting the environment (Helm, 2020). The pandemic has forced us to reassess priorities, emphasize distinctions between needs and wants, and alter consumer behavior (Sheth, 2020). Creative expression, especially in music and film, has also been modified through low budget productions by artists in their homes. The adage of *less is more* is seeming to be verified in real time, owing to the effects of resource scarcity – but not necessarily time constraints – on creative output (Mehta and Zhu, 2016).

As we move forward, the ideal situation would allow us to have the best of both worlds—the connections that we had lost as well as the new skills we have gained. The way the pandemic will have shaped our future behavior will remain unknown for the time being. Until then, we can choose to focus on our creative present, keeping somewhere in our minds all of the potential ramifications.

## Data Availability Statement

The original contributions presented in the study are included in the article. Further inquiries can be directed to the corresponding author.

## Author Contributions

HK: conceptualization, resources, writing – original draft, and writing – review and editing. JK: conceptualization, writing – original draft, and writing – review and editing. Both authors contributed to the article and approved the submitted version.

## Conflict of Interest

The authors declare that the research was conducted in the absence of any commercial or financial relationships that could be construed as a potential conflict of interest.

## Footnotes

1 https://bit.ly/2WzFGJN

2 https://bit.ly/3jojBHV

3 https://bit.ly/2P4OseR

4 https://www.safelymakingca.org/
